# The inactivation of Ascaris suum eggs by short exposure to high temperatures

**DOI:** 10.2166/washdev.2018.051

**Published:** 2018-12-10

**Authors:** D. Naidoo, C. C. Appleton, C. E. Archer, G. L. Foutch

**Affiliations:** 1 **D. Naidoo** (corresponding author) **C. E. Archer** Pollution Research Group, Howard Campus, University of KwaZulu-Natal, Durban, South Africa; 2 **C. C. Appleton** School of Life Sciences, Westville Campus, University of KwaZulu-Natal, Durban, South Africa; 3 **G. L. Foutch** School of Computing and Engineering, University of Missouri Kansas City, 64110, USA and Chemical Engineering, Howard Campus, University of KwaZulu-Natal, Durban, 4001, South Africa

**Keywords:** *Ascaris suum*, heat treatment, inactivation, temperature, viability

## Abstract

*Ascaris* sp. is the most prominent and resilient helminth of human health importance found in faecal sludge, making *Ascaris* sp. an ideal index organism for inactivation testing. Heat treatment destroys helminths,allowingfor safe handling and possible reuse of sludge. Technology developmentfocuses on rapid heating to minimize equipment size and cost. This study evaluates *Ascaris suum eggs'* viability with short heating time. *Ascaris eggs* were placed in a water bath at temperatures from 60 to 80 °C for various exposure times (5 seconds to 4 minutes) and were immediately processed and analysed via light microscopy. For all samples within these temperature and time ranges, less than 10% viable eggs were recovered. For 70, 75 and 80 °C, complete inactivation was observed for exposure time of 5 seconds and above.

## INTRODUCTION

Approximately 2.3 billion people globally, and 695 million of the sub-Saharan Africa population, do not have access to improved sanitation (JMP 2017; Roche et al. 2017), with an estimated 1 billion performing open defecation (Tiwari et al. 2017). Approximately 2 billion people globally are infected with soil-transmitted helminths (Yadav & Mahato 2017). The inability of countries to provide water, sanitation and hygiene (WASH) results in 1.3 million deaths annually due to diarrhoeal diseases. Diarrhoeal disease is the cause of one in eight child mortality cases in children under five years of age worldwide (Kotloff 2017).

Diarrhoea manifests generally as a symptom of bacterial and viral infections but may also be a symptom of infection

This is an Open Access article distributed under the terms of the Creative Commons Attribution Licence (CC BY 4.0), which permits copying, adaptation and redistribution, provided the original work is properly cited (http://creativecommons.org/licenses/by/4.0/ ). doi: 10.2166/washdev.2018.051 by parasitic worms (helminths), with Ascaris lumbricoides of greatest concern (Brownell & Nelson 2006). Ascaris eggs are the most resilient of all organisms found in sludge, as they can withstand harsh environmental conditions, such as desiccation, and can survive in both aerobic and anaerobic environments for up to seven years in the soil (Pecson & Nelson 2005). Ascaris spp. are therefore deemed fit as index organisms for parasitic contamination, as well as overall pathogenic contamination and inactivation (Sidhu & Toze 2009). If a treatment process can inactivate Ascaris spp. eggs, then it is very likely all other pathogens will be destroyed as well (Maya et al. 2012). Ethical approval for the collection of stool samples from human hosts (to obtain the human roundworm, Ascaris lumbricoides) is difficult. Ascaris suum, a parasite of pigs, is therefore sometimes used as a surrogate for A. lumbricoides, as the two species are morphologically indistinguishable from one another in all stages (Daugschies et al. 2013). A. suum eggs were therefore used in this study.

Ventilated pit latrines (VIP) are regarded as improved sanitation by the South African government. Once full, the pits are sealed or emptied (Bhagwan et al. 2008). Proper dis-posal and treatment of human excreta is important in reducing transmission of infectious diseases. Land application of treated sludge is becoming increasingly common, thereby encouraging resource reuse and recovery, alleviating environmental contamination and reducing human health impacts (Fewtrell & Bartram 2001).

Increased temperatures have been found to be effective and cost efficient for inactivation of faecal-sludge pathogens (Podichetty et al. 2014; Belcher et al. 2015). This study is an extension of a short communication by Naidoo & Foutch (2017) and aimed to fill the knowledge gap with regards to the effects of short exposures of Ascaris eggs to heat. It was part of a larger project funded by the Bill and Melinda Gates Foundation (BMGF), which focused on development of a prototype viscous heater for sludge treatment by heat exposure. Thomas et al. (2015) focused on a range of temperatures at exposure times >10 minutes. Brannen et al. (1975) and Thomas et al. (2015) reported Ascaris inactivation at 64 and 70 °C, respectively, for 1 minute exposure. No studies focus on inactivation of Ascaris eggs for exposure times less than a minute (Thomas et al. 2015). Test parameters for this study ranged from 60 to 80 °C, at exposure times of 5 seconds to 4 minutes, simulating the effects of the viscous heater during treatment. The isolated effects of heat on Ascaris eggs in water was investigated, rather than sludge, which might serve as a matrix to protect eggs against heat exposure.

## MATERIALS AND METHODS

A. suum eggs were purchased from Excelsior Sentinel Inc. (USA) and stored at 4 °C until needed. Egg stock solutions were prepared from the initial egg samples with approxi-mately 550 eggs per 1 mL of solution. A 34-litre heating water bath controlled experimental exposure temperatures. Eggs were exposed to heat treatments in 15 mL polypropy-lene test tubes preheated in the water bath by adding 13 mL of boiling water per tube and immersing these tubes into the water via a modified polystyrene rack. A thermocouple and data logger monitored temperature over time. Eggs were analysed via light microscopy, before and after incubation at 25 °C for 28 days in a cooling incubator.

**Figure 1:**
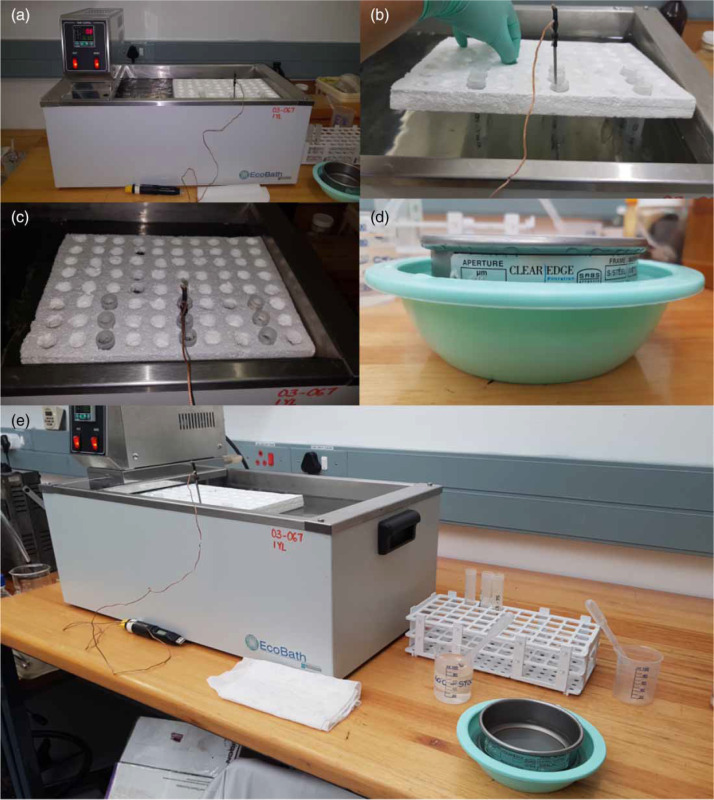
Experimental setup for heat treatment. (a) and (e) Preheated water bath with polystyrene racks containing test tubes and thermocouple. (b) and (c) Polystyrene rack setup with tubes containing water samples with spiked eggs in 15 mL plastic test tubes. (d) Sieve immersed in tap water, in plastic bowl.

Experimental temperatures were 60, 65, 70, 75 and 80 °C, and exposure times 5, 10, 15, 30 and 45 seconds, and 1, 2, 3 and 4 minutes. For 80 ^°^C, shorter exposure times of 1-10 seconds were also tested. The water bath was preheated prior to each experiment. The thermocouple was placed into the tube prior to exposure to ensure steady-state temperature (Figure 1). For each treatment, a 1 mL aliquot of egg stock solution was placed into a 15 mL test tube and used to assess developmental states of the eggs before exposure to heat. This allowed counting of the total number of eggs per 1 mL of solution. One mL aliquots of egg stock solutions (approximately 550 eggs per 1 mL) were pipetted into the test tubes using a 1 mL Pasteur pipette and time recorded as T0. Eggs were exposed for the respective times, after which the tube was immediately removed from the water bath. Samples were returned to room temperature to avoid prolonged heat exposure, by emptying the contents of the test tube (i) into beakers con-taining 40-50 mL iced water at approximately 9 °C or (ii) onto a 20 |im sieve within a bowl containing tap water at room temperature (Figure 1(d)). Control samples were not heated but were cooled and processed similarly to treated samples.

Eggs were processed following Moodley et al. (2008) and Pebsworth et al. (2012), modified by Naidoo et al. (2016). Ammonium bicarbonate (made up with deionized water to form 99% ammonium bicarbonate, NH4HCO3 by Sigma) was used to rinse the test tubes and beakers that previously contained treated samples. The tube or beaker contents were rinsed thoroughly over a 20 |im sieve. The deposit was transferred into a plastic 15 mL test tube using a Pasteur pipette and centrifuged at 1,310 x g (3,000 rpm) for 5 minutes using a bench-top centrifuge (with a swing-out rotor). The supernatant was discarded, and the egg pellet was analysed under a light microscope at 100x and 400x magnification.

Egg viability was assessed by pipetting two drops of egg suspension onto a plain glass microscope slide and covering with a 40 x 22 mm coverslip. The eggs were categorized as either potentially viable or non-viable based on morphology.

Potentially viable (PV) eggs included those that were unde-veloped (at different cell stages), embryonated with a visibly motile larva, and embryonated containing an immotile larva (Naidoo et al. 2016). Non-viable (NV) eggs included those that were dead (globules inside the egg shell), embryonated with a necrotic larva, and mechanically broken (Naidoo et al. 2016). Samples were analysed before and after incubation such that treated samples were immedi-ately analysed, washed back into the test tube after counting, incubated for 28 days at 25 °C and then re-analysed after incubation. This process was repeated for all temperatures and exposure times and for controls. Eggs were further analysed via oil immersion microscopy (1,000 x, 1,500x and 2,000x) in order to evaluate heat damage in greater detail (three-gear focusing using a magnification changer allowed for magnifications greater than 1,000x).

Normality of data was tested using a one-sample Kolmo-gorov-Smirnov test using IBM SPSS Statistics (version 24, IBM Corp., Armonk, NY, USA) and transformed accord-ingly using arcsine transformation, then analysed statistically. A nested analysis of variance (ANOVA) was run on the data using R (version 3.0.2, R Core Team 2016). The Shapiro-Wilk test was used to test for normality and a Levene's test was used to test for homogeneity of variance of the studentized residuals from the ANOVA. Tukey's multiple comparisons post-hoc test was used to compare means of egg viability across and between different treatments. Percentage viability was calculated as follows:

Percentage viable eggs recovered =(Total viable eggs recovered/Total viable eggs inoculated) *100(1)

An independent samples t-test was run on percentage viable eggs recovered, comparing processing with iced or tap water. A second independent samples t-test was run on percentage of viable eggs recovered before and after incubation for each replicate of each treatment (IBM SPSS v.24). The criterion set for this study for successful inactivation was < < 10% viable eggs recovered after treatment. This is similar to a study by Aycjgek et al. (2001) and Naidoo et al. (2016), which considered 90% mortality of eggs as significant.

## RESULTS

Eggs that appeared undamaged and remained at the one-celled stage after treatment were scored as undeveloped pre-incubation, as embryo death could only be confirmed post-incubation. The percentage of viable eggs recovered pre-incubation is therefore much greater than post-incubation, especially for treatment combinations that did not yield visible egg damage. The p-values are not only indicatore of significant differences between percentage of viable eggs recovered, but also the level of damage and the general morphological states between exposure times. Samples exposed for 5 seconds at 70, 75 and 80 °C resulted in the most significant shift in egg state, post-incubation (from undeveloped to developed) and were used as the base-line for comparison of successive exposure times (the least damage could be seen after 5 seconds). The general ANOVA (Table 1) indicated that temperature, independently, and in conjunction with secondary variables such as exposure time, processing with iced or tap water and point of analysis, had a significant effect on percentage of viable eggs recovered (p < 0.001).

**Table 1:** Statistical results of the general ANOVA from the nested ANOVA design, comparing the percentage of viable eggs recovered between each tested exposure time to 80 °C

Primary variable	F_(df)_	p-Value	Variable combinations^a^	p-Value
Temperature	F(_6>_ 552)=i705,8	<0.001	Temperature/Exposure time	<0.001
			Temperature/Exposure time/Processing	<0.001
			Temperature/Exposure time/Processing/Analysis	<0.001

a Variable combinations represent the nestedness of the statistical design. Processing refers to the method used (iced or tap water) and analysis refers to whether eggs were examined before or after incubation.

The first level of nestedness compares each temperature (primary variable) against other temperatures and two controls for the percentage of viable eggs recovered after treatment. No significant difference was observed between control 1 (iced water) and control 2 (tap water) (p — 0.760), indicating that the processing method had no effect on percentage viability (note: controls were not heat treated but were processed by the same method as treated samples). There was, however, a significant difference in viable eggs recovered between each test temperature combination from 60 °Cto 80 °C(p < 0.001), as well as between controls and heat-treated samples (p< 0.001).

**Figure 2:**
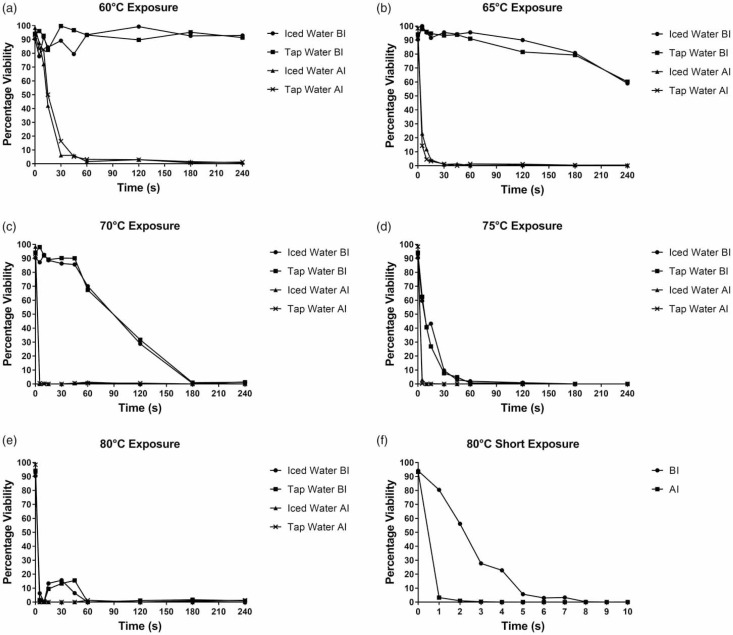
Percentage of viable eggs recovered after treatment at 60 to 80 °C for 5 seconds to 4 minutes (a)–(e), and 1 to 10 seconds (f), processed with either tap or iced water, pre- and post-incubation at 25 °C for 28 days (*n* = 3). There was no significant difference between processing method (*p* > 0.05), thus only iced water was used to process very short exposure samples (1 to 10 seconds) at 80 °C. BI represents percentage of viable eggs recovered before incubation and AI represents percentage of viable eggs recovered after incubation.

The second nested level compares the effect of each test temperature (primary variable) on the percentage of viable eggs recovered after treatment across various exposure times (secondary variable). Samples were compared across temperatures at each exposure time. When comparing the controls with 60 °C, no significant difference in development was seen up to 15 seconds (p > 0.05), however exposure times of 30 seconds and above showed a significant decline in development (Figure 2(a)). There was a significant difference in development up to 15 seconds ( p < 0.001) between 60 and 65 °C (comparing Figure 2(a) and 2(b)). From 30 seconds onwards, there were no significant differences between each exposure time (p > 0.05), and a similar trend was seen for 65 °C. Viable egg recovery had dropped to 0% after 4 minutes' exposure, although at 60 °C, minimal development was still evident. At 60 °C, per-centages of viable eggs recovered post-incubation were mostly <10% per treatment, and almost 0% at 3 and 4 minutes (Figure 2(a)). Little visible egg damage was evident preincubation, thus they were initially classified as viable (unde-veloped eggs). When considered together with the low viability percentages shown in Figure 2(a), successful inacti-vation at 60 °C can be attributed to exposures of >45 seconds, which met the criterion for this study.

Significant egg viability differences were observed between 5 seconds and every time interval from 10 seconds to 4 minutes (5:10 seconds, p — 0.002; 5:15 seconds up to 4 minutes, p < 0.001), indicating an increase in visible damage after 5 seconds when exposed to 65 °C(Figure 2(b)). Between exposures of 5 and 10 seconds, the percentage of viable eggs dropped to approximately 10% (post-incubation), and almost zero after 15 seconds onwards. There was no significant change in the state of eggs after treatment from 10 seconds onwards and between each time interval(p > 0.05), confirming that successful inactivation (<10°/o viable eggs recovered) occurred after exposures of 10 seconds and longer, meeting the criterion of this study. Complete die-off was, however, not achieved fully for either 60 or 65 °C as isolated cases of egg development were evident even after 4 minutes of exposure.

When comparing across temperatures between 60 and 70 °C, 75 and 80 °C, significant differences in egg viability were noted at all test exposure times, indicating a decline in egg development (thus increased effectiveness of treatment of the eggs) for each successive exposure time (Figure 2(a)-2(e)). The same can be said for comparisons between 65 and 75 °C and between 65 and 80 °C exposures (p < 0.001). As seen in Figure 2(c), visible damage occurred only at 2 minutes' exposure to 70 °C, however significant die-off occurred from 5 seconds' exposure onwards. A similar level of damage to the eggs occurred after treatment at 70 ^°^C across all exposure times (p > 0.05 between each time interval). At 75 °C, asignifi-cant difference in egg viability was observed between 5 seconds and all subsequent exposure times from 10 seconds to 4 minutes (p < 0.001), indicating an increase in visible damage from >10 seconds' exposure (Figure 2(d)).

No significant differences in egg viability were observed between 5 seconds and all other exposure times from 10 seconds to 4 minutes (p > 0.05), indicating that visible damage occurred from the first exposure time point (5 seconds) when exposed to 80 ^°^C(Figure 2(e)). Further analy-sis of exposure to 80 °C for shorter periods (1-10 seconds) showed that visible damage to the eggs was only apparent after exposures of 4-5 seconds (Figure 2(f)). Although eggs appeared healthy and undamaged after exposure to 80 ^°^C for 1 -10 seconds, most failed to develop further after incubation. Some development was evident for eggs exposed for up to 2 seconds. Even so, development was arrested at stages of cleavage between two cells and the gastrula. From 5 seconds and longer at 70, 75 and 80 ^°^C, little to no further development was observed in eggs post-incubation, indicating successful inactivation, meeting the 10/ criterion set for this study (Figure 2(c)-2(e)).

Results from the independent samples t-test between the two processing methods (cooling by iced and tap water) indicated no significant difference in the percentage of viable eggs recovered after treatment (p > 0.05). The third level of comparison of the nested ANOVA (temperature/ exposure time/processing method) was therefore not out-lined in further detail.

## DISCUSSION

Table 2 summarizes the results from the inactivation trials and indicates which exposure time was most effective in inactivating eggs for each test temperature. It highlights the exposure time at which damage occurred but was not visible (where further development did not occur post-incubation), visible damage occurred (globular eggs seen as indicated by Naidoo et al. (2016) to be explored in a second article) and complete die-off was achieved (where no viable eggs were recovered after treatment). For each column, a decreasing trend can be observed, indicating that with increasing temperature, the time required for damage (both non-visible and visible) and egg inactivation to occur, decreases. For 60 and 65 °C, die-off did not reach a 0% level, with few eggs able to develop, although viability percentages after incubation were negligible.

**Table 2:** Summary of the *Ascaris* egg viability results for each test temperature of the current study

Temperature (°C)	Damage not visible	Visible damage	Complete die-off^b^
60°C	30 seconds	3 minutes	–
65°C	15 seconds	3 minutes	–
70°C	5 seconds	2 minutes	15 seconds
75°C	5 seconds	1 minute	10 seconds
80°C	5 seconds	5 seconds	5 seconds
80°C – Short^a^	1 second	4 seconds	4 seconds

a Short refers to the repeated 80 _C experiment with exposure times between 1 and 10 seconds.

b Complete die-off was confirmed by microscopic analysis post-incubation.

The current study indicated that heat treatment is a suc-cessful inactivation method when applied over a range of temperatures. This study also contributes towards filling the literature gap that exists for short temperature exposures and the resulting effect on the viability of Ascaris eggs. Exposure to each of the test temperatures was successful on its own, and eventually reached either negligible viability percentages (60 and 65 ^°^C) or complete die-off (0% viability at 70, 75 and 80 °C), after exposure for up to 4 minutes.

As already mentioned, exposures at 60 and 65 °C resulted in almost 100% inactivation, except for a few eggs that developed after incubation. Popat et al. (2010) investigated inactivation of A. suum by anaerobic digestion at thermophilic temperatures (thermophilic refers to the ability of an organism, in this case bacteria, to survive temperatures ranging from 46 to 108 °C). It was reported that there was a reduction in egg viability after 2 hours of heat treatment at 51.1-55 °C and that the composition of the sludge might play a role in the effectiveness of heating.

Brannen et al. (1975) and Brandon (1978) both investi-gated the effects of heat exposure on the embryonation of Ascaris lumbricoides eggs, in a controlled-temperature water bath, similar to that used in the current study. Bran-nen et al. (1975) reported a near complete inhibition of egg development when exposed to 55 ^°^C for 4 minutes and <1% embryonation of eggs when exposed to 64 °C for 1 minute. Brandon (1978) reported that 1 hour of exposure to 55 °C was sufficient for reducing the number of viable Ascaris eggs to near negligible amounts. Both studies support the findings of the current study, but only at the lower test temperatures, showing a similar relationship between exposure to heat and egg inactivation (Figure 2).

Steer & Windt (1978) investigated the effects of compost-ing on Ascaris eggs. They concluded that temperatures of 65 °C should be maintained for 70 days within the compost-ing system for successful inactivation of eggs. The results of the current study challenge the recommendations of Steer & Windt (1978), as exposure to 65 °C for 30 seconds was suffi-cient for negligible inactivation percentages (Table 2 and Figure 2(b)). It can be argued that the different methods of heat exposure in the two studies resulted in the discrepancy between the reported inactivation rates.

Thomas et al. (2015) investigated the relationship between A. suum egg inactivation and the effects of heat and shear stress/force independently and combined. The methods for testing heat treatment were similar to the cur-rent study. Human-faecal simulants were spiked with A. suum eggs and smeared along the inside of 7 mL poly-ethylene vials (Thomas et al. 2015). The vials were sealed and wrapped in Parafilm to prevent water entering, and were fully submerged in a water bath for 60 seconds at 40 °C. Treatments increased in 10 °C intervals up to 100 ^°^C. Cooling to room temperature was done by immersing the heated vials in a 27 °C water bath for 60 seconds. At 47, 51 and 55 °C, the embryonation rates of eggs were 94.7, 91.1 and 89.7%, respectively. This aligns with the current study, which found that eggs were able to continue development at temperatures up to 65 °C when exposed to heat for up to 2 minutes (viability is visible in Figure 2(b), even if negligible). Low temperature treatment therefore requires longer exposure times.

At 70 °C, 5 seconds' exposure was sufficient to halt further embryo development, with 100% inactivation occur-ring from 15 seconds onwards. Thomas et al. (2015) stated that for temperatures of 70 °C and above, almost no viable eggs were recovered after treatment. This supports the data presented in the current study, where egg development, if any, at temperatures from 70 °C onwards, approached zero viable egg recovery (Figure 2(c)). Belcher et al. (2015) published the first study on Ascaris eggs based on the viscous heater, that serves as a preamble to the current study which provides important foundation work on the effects of temperature, independently, on Ascaris eggs. A baseline for egg inactivation was determined by feeding seeded sludge into the heater. Representative samples were taken for each inlet pump and rotating core speed combination, at increasing and plateau temperatures, in order to determine egg viability. The operational speeds also determined residence time for specific generated temperatures. The findings of Belcher et al. (2015) were in line with Thomas et al. (2015), by recording 90% egg inactivation at temperatures above 70° C, further supporting the findings of the current study. At 75 and 80 °C and <10 seconds of heat exposure, 100% egg inactivation was achieved (Figure 2(d)-2(f)). In most cases, all structural and morphological integrity of both the egg and the contained embryo were lost. Belcher et al. (2015) also reported 100% egg inactivation when exposed to 85 °C with residence times under 1 minute, which was in line with the present study's findings of 100% inactivation at 80 °C for 4 seconds.

Pecson & Nelson (2003) investigated the effects of temperature, exposure time, pH and ammonia on the inactivation of Ascaris eggs. Eggs were stored at different pH levels, ammonia concentrations and temperatures for 24 hours. Between a threshold temperature of 44 to 48 °C, eggs went from low to complete inactivation. In the absence of ammo-nia, 99% inactivation was reported at 48 °C and pH 7, 9 and 11. When the ammonia concentration was held Constant at 1,000 ppm with pH 7 at 48 °C, pH 9 at 44 °C and pH 11 at 44 °C, 99% inactivation was achieved. At lower pH values, the presence of ammonia had a negligible effect on inactivation rates, as a temperature of 48 °C was still required for 99% inactivation. At higher pH values, the presence of ammonia reduced the temperature required for 99% inacti-vation, indicating that the addition of ammonia is a valuable secondary treatment option in combination with temperature. This is because the alteration of eggshell per-meability by heat may allow for easier penetration of the ammonia (or possibly other chemical treatments) (Barrett 1976).

## CONCLUSIONS AND RECOMMENDATIONS

- All test temperatures (between 5 seconds and 4 minutes) met the criterion for this study, i.e., inactivation was considered successful if the recovery of viable eggs after treatment and incubation was <10% (Figure 2(a)-2(f)).
- Complete die-off within the tested exposure time range was noted for 70 °C, 75 °C and 80 °C, however treatment at 60 °C and 65 °C allowed for development of a few eggs after incubation.
- Incubation of heat-treated samples is required when egg damage is not visible, in order to confirm die-off.
- The results of the current study therefore show that residence times of as low as 4 seconds at 80 °C may be recommended for successful inactivation when using the viscous heater.
- Field application of treatment processes, as well as treatment in the indigenous medium and not a saline suspension may result in differing inactivation levels. As explained by Jebri *et al.* (2013) and Mun et al. (2009), the suspension medium of the eggs determines the extent of heat treatment. Buttar *et al.* (2013) reported that sludge or any similar faecal simulant may act as insulation for eggs exposed to heat, suggesting that higher temperatures and prolonged exposure times might be needed for testing eggs in vitro. The role of the suspension medium during heating needs to be investigated. Further work is also required to test the actual effects of heat treatment of the eggs using the viscous heater.
